# α-Defensin HD5 Stabilizes *Human Papillomavirus 16* Capsid/Core Interactions

**DOI:** 10.20411/pai.v4i2.314

**Published:** 2019-09-12

**Authors:** Neetu M. Gulati, Masaru Miyagi, Mayim E. Wiens, Jason G. Smith, Phoebe L. Stewart

**Affiliations:** 1 Department of Pharmacology, Case Western Reserve University, Cleveland, Ohio; 2 Cleveland Center for Membrane and Structural Biology, Case Western Reserve University, Cleveland, Ohio; 3 Department of Microbiology, University of Washington, Seattle, Washington

**Keywords:** alpha-Defensins, Human papillomavirus 16, Cryo-electron Microscopy, Mass Spectrometry, Molecular Dynamics, Intrinsically Disordered Proteins, Virus Host Interactions

## Abstract

**Background:**

*Human papillomavirus* (HPV) is linked to nearly all cases of cervical cancer. Despite available vaccines, a deeper understanding of the immune response to HPV is needed. Human α-defensin 5 (HD5), an innate immune effector peptide, blocks infection of multiple sero-types of HPV, including high-risk HPV16. While a common mechanism of α-defensin anti-viral activity against nonenveloped viruses such as HPV has emerged, there is limited understanding of how α-defensins bind to viral capsids to block infection.

**Methods:**

We have used cryo-electron microscopy (cryoEM), mass spectrometry (MS) crosslinking and differential lysine modification studies, and molecular dynamics (MD) simulations to probe the interaction of HPV16 pseudovirions (PsVs) with HD5.

**Results:**

CryoEM single particle reconstruction did not reveal HD5 density on the capsid surface. Rather, increased density was observed under the capsid shell in the presence of HD5. MS studies indicate that HD5 binds near the L1 and L2 capsid proteins and specifically near the C-terminal region of L1. MD simulations indicate that favorable electrostatic interactions can be formed between HD5 and the L1 C-terminal tail.

**Conclusions:**

A model is presented for how HD5 affects HPV16 structure and cell entry. In this model, HD5 binds to disordered regions of L1 and L2 protruding from the icosahedrally ordered capsid. HD5 acts to cement interactions between L1 and L2 and leads to a closer association of the L2/genome core with the L1 capsid. This model provides a structural rationale for our prior observation that HD5 interferes with the separation of L1 from the L2/genome complex during cell entry.

**Graphical Abstract:**

**Figure:**
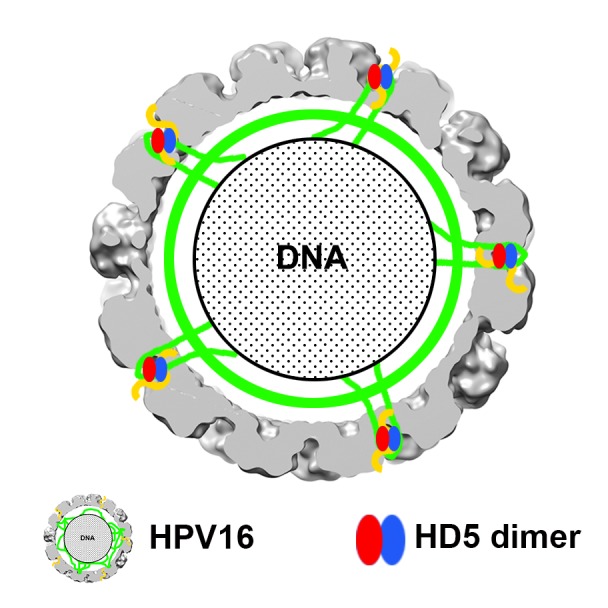

## INTRODUCTION

*Human papillomavirus* (HPV) infections are a major health burden. HPV is the most common sexually transmitted infection, causing a range of conditions including genital warts and cancer. While most HPV infections do not cause major health concerns, persistent genital HPV infection is linked to nearly all cases of cervical cancer, in addition to other types of cancers in men and women [1, 2]. Worldwide, cervical cancer causes approximately 270,000 deaths annually, with the highest mortality rates in low- and middle-income countries [3]. Vaccines against certain strains of HPV, including those that cause cervical cancer and genital warts, are clinically available [4]. They are believed to function primarily by eliciting neutralizing antibodies, and the humoral response to HPV infection has been an area of intense research [5, 6]. However, recognition of HPV by the innate immune response and the role of innate responses in stimulating a potent adaptive response to HPV is less well understood [7].

Defensin antimicrobial peptides are effector components of innate immunity. They are directly antimicrobial but also function indirectly through immunostimulatory activity [8-12]. Both of these activities likely contribute to their role in host defense against bacterial, viral, and fungal infections. Human defensins are classified into 2 groups, α and β, based on structure and sequence. The α-defensins are further subdivided into myeloid and enteric, based on the cell types in which they are primarily expressed. We have focused on the activity of human α-defensin 5 (HD5), because it is expressed in the male and female genitourinary tract [13-15] and has been shown to potently block infection of multiple serotypes of HPV, including high-risk HPV16 [16-20]. HPV16 and HPV18 together cause about 70% of all cervical cancers and are the 2 highest risk types of HPV [1].

A broad mechanism of α-defensin neutralization of non-enveloped viruses has emerged from studies of adenoviruses, polyomaviruses, and papillomaviruses [8, 19]. Although they do not block and have in some cases been shown to enhance initial binding of viruses to cells, α-defen-sins perturb uncoating and prevent the DNA genomes of these viruses from reaching the nucleus. For adenoviruses, HD5 bridges the interaction between 2 capsid proteins, thereby stabilizing the viral capsid and completely blocking uncoating within the endosome [21-25]. JC polyomaviruses are also neutralized by HD5-induced viral stability, which leads to altered intracellular trafficking [26]. The mechanism of HPV16 neutralization is more complex in that there are at least 2 effects of HD5 on entry. First, cleavage of the minor capsid protein L2 at the cell surface is blocked [18]. Second, although the virus is internalized and partially uncoats, the genome remains aberrantly associated with the major L1 capsid protein and fails to traffic to the nucleus [19]. The only exception to this paradigm of viral stabilization is that HD5 and the myeloid α-defensin HNP1 inhibit BK polyomavirus primarily by aggregating the virus and preventing binding to host cells [27]. Although all of these antiviral functions rely on defensin binding to the capsid, the molecular features recognized by α-defensins and their localization on the capsids of these disparate viruses are largely unknown.

To address this gap in knowledge and to clarify the structural mechanism of HD5 neutralization of HPV, we have used cryo-electron microscopy (cryoEM) and mass spectrometry to investigate the effect of HD5 binding on the HPV16 structure. The HPV capsid is composed of 72 L1 pentamers, arranged so that some pentamers have 5 neighbors (pentavalent capsomers) and others have 6 neighbors (hexavalent capsomers), and an undefined copy number of L2. The capsid is stabilized by disulfide bonds between cysteine residues of neighboring L1 capsomers [28], and a recent near-atomic resolution cryoEM structure of HPV has provided pseudoatomic coordinates for the L1 portion of the HPV16 capsid (PDB: 5KEP) [29]. The HPV L2 protein co-assembles with L1 and serves multiple functions including DNA packaging [30, 31], facilitating escape of the L2/genome complex from late endosomes and trafficking of the genome during entry [32-37], and chromatin binding linked to translocation of the L2/genome complex across the limiting cellular membrane during mitosis [38, 39]. L2 is predicted, for the most part, to be an intrinsically disordered protein [38], and it is also mostly an internal capsid protein [40]; however, there are regions of L2 that are known to emerge from the virion during the cell entry process and become accessible on the capsid surface [41-43]. Consistent with these observations, Guan *et al* tentatively assigned diffuse density on the exterior capsid surface primarily on the hexavalent L1 capsomers to L2 [29]. However, the precise locations of L2 within the capsid remain unclear.

In contrast to our studies of human adenovirus serotype 5 in complex with HD5 [23, 25], density attributable to HD5 on the HPV16 capsid is not readily apparent by cryoEM. Rather, the most notable structural change in HPV16, when in complex with HD5, is the increased ordering of density immediately below the capsid. Mapping by mass spectrometry provides evidence for HD5 interactions with disordered or flexible regions of L1 and L2. Molecular dynamics (MD) simulations indicate that favorable electrostatic interactions can be formed between the L1 C-terminal tail and an HD5 dimer. Thus, the combination of cryoEM, mass spectrometry, and MD simulations suggest a mechanism whereby HD5 binding entangles L1 and L2 by cementing interactions between disordered regions, leading to a more regular association between the genome and the capsid in the presence of HD5. This finding explains the failure of L1 and L2 to dissociate during cell entry in the presence of HD5 [19].

## METHODS

### HPV16 Pseudovirus Production

HPV16 pseudovirus (HPV PsV) containing the major capsid protein L1 and minor capsid protein L2 were prepared as previously described [44-47]. Briefly, an HPV16 PsV seed stock was made by co-transfecting 293TT cells with plasmids encoding codon-optimized HPV16 L1 and L2 (p16L1L2, gift of Martin Muller, GCRC) and an eGFP reporter (pfwB, gift from John Schiller, NCI). Cleared lysate from this transfection contained mature PsV and was then used to infect additional 293TT cells. The cells were lysed, and ammonium sulfate was added to a final concentration of 25mM to improve maturation of the HPV16 PsV. The lysate was treated with benzonase (SigmaAldrich) and Plasmid Safe RNase (Epicentre). NaCl was added to a final concentration of 850mM, and the HPV16 PsV was purified by ultracentrifugation through an OptiPrep gradient. OptiPrep was removed from the HPV16 PsV via agarose gel filtration, and buffer was exchanged into Dulbecco's phosphate buffered saline (DPBS) with 0.8M NaCl.

### CryoEM Grid Preparation

In preparation for cryoEM studies, the purified PsV sample (~400 ng/µL) was diluted 75-fold in DPBS with 0.5M NaCl at pH 7.4. A 19.5 µL sample of diluted PsV was combined with 0.5 µL of concentrated HD5 (400µM in water) to produce a mixture with a final concentration of 10µM HD5, which is sufficient to neutralize HPV16 under these conditions. For these experiments, synthesized linear HD5 peptide (CPC Scientific, Sunnyvale, CA) was subjected to thiol-disulfide reshuffling and purified to homogeneity by reverse-phase high-pressure liquid chromatography to generate folded HD5 [20]. The PsV/HD5 mixture was incubated on ice for 45 minutes, and 3 µL HPV16 PsV alone or HPV16 PsV with HD5 was then applied to glow-discharged Quantifoil 2 × 2 400 mesh holey carbon grids. Grids were blotted until nearly dry and rapidly frozen in liquid ethane using a manual plunger.

### CryoEM Imaging and Data Collection

CryoEM micrographs were collected on an FEI Titan Krios 300 kV transmission electron microscope with a Direct Electron DE20 direct detector for a total electron dose of 6000 electrons/nm^2^. Micrographs were collected at 29,000 × magnification with a defocus range of 0.8 µm to 3.5 µm. The HPV16 PsV alone dataset included 3304 micrographs and the HPV16 PsV with HD5 dataset included 3868 micrographs. Frame alignment and radiation dose damage compensation of the micrographs was performed with the DE_process_frames software (Direct Electron).

### Particle Picking and CTF Correction

Individual particles were selected in a semi-automated fashion using E2 Boxer in the EMAN2 software package [48]. Estimations of the defocus values for the micrographs were made using GCTF [49].

### 3-D Structure Determination and Filtering

RELION 2.0 was used to extract individual particles from micrographs (0.126 nm/pixel or binned data at 0.252 nm/pixel) and for subsequent steps in refinement. A cryoEM structure of the HPV16 PsV (EMD: 5932) filtered to 6 nm resolution was used as the initial model for 3-D reconstruction [47]. This model was used to determine the orientation of each particle. A subset of particles was chosen through 2-D classification and 3-D classification for each structure. Binned data (0.252 nm/pixel) was used for 2-D classification and unbinned data (0.126 nm/pixel) was used for 3-D classification and refinement. Final refinement of the maps used 2900 particles for HPV16 PsV alone and 1742 particles for HPV16 PsV with HD5 using RELION 2.1. After refinement and postprocessing, the final resolutions of the HPV16 alone and HPV16+HD5 maps were 0.53 nm and 0.49 nm, respectively, as measured at the 0.143 threshold of a gold standard Fourier Shell Correlation plot. EMAN2 was used to low-pass filter the HPV maps to 2 nm resolution [48].

### Chemical Crosslinking of HPV16 PsV and HD5

The complex of HPV16 PsV (1 µL containing 400 ng) and HD5-R13K/R32K [20] (2 µL containing 200 pmol) was mixed with 8μM bissulfosuccinimidyl suberate (BS3) and incubated at 25°C for 45 minutes in 10 µL of DPBS. The reaction was stopped by adding 1 µL of 130mM Tris buffer, pH 7.4. The reaction mixture was separated on a NuPAGE 4%–12% Bis–Tris gel using MES running buffer, and the crosslinked protein complexes were visualized by SYPRO Ruby. Three regions of the SDS-PAGE gel corresponding to crosslinked complexes for L1-HD5, L2-HD5, and the L1-L2 dimer (Figure S2) were excised, the proteins were in-gel digested using trypsin [50], and the digests were analyzed by LC-MS/MS as described below. Possible crosslinked peptides were identified using MassMatrix database search software (Version 3.10, MassMatrix, Columbus, OH), and all the possible crosslinked peptides were manually verified by inspecting their MS/MS spectra.

### Differential Lysine Modification

HPV16 PsV alone (1 µL containing 400 ng) and HPV16 PsV complexed with HD5 (2 µL containing 200 pmol) were treated with unlabeled- and ^13^C_4_-labeled-acetic anhydride (1 µL containing 100 nmol), respectively, in 10 µL of DPBS at 25°C for 1 hour. We also carried out a reverse experiment where the ^13^C_4_-labeled acetic anhydride was used to modify the HPV16 PsV alone sample and unlabeled acetic anhydride to modify the HPV16 PsV-HD5 complex sample. After the reaction, 2 μL of 1M hydroxylamine in water was added to the reaction mixture and incubated at 25°C for 30 minutes to stop the reaction as well as to reverse the acylation on tyrosine, cysteine, and histidine that might have occurred. Then, the samples treated with labeled and unlabeled acetic anhydride were mixed, transferred into an Amicon Ultra-0.5 10K centrifugal filter device, and the buffer salts, the reagents, and Optiprep in the sample were removed by repeated addition of 8M urea in 100mM ammonium bicarbonate and centrifugation at 14,000*g*. The proteins were reduced by dithiothreitol (DTT) and S-alkylated by iodoacetamide; then the urea concentration was reduced to < 2M and the proteins digested by trypsin in the filter device. The digest was analyzed by LC-MS/MS as described below. Lysine-acetylated peptides were identified using MassMatrix database search software. Carbamidomethylation of cysteine was set as a fixed modification, whereas variable modifications included ^12^C_2_-acetyl-lysine, ^13^C_2_-acetyl-lysine, and oxidation of methionine to methionine sulfoxide. The mass tolerance was set at 10 ppm for precursor ions and 0.8 Da for product ions. Strict trypsin specificity was applied. The ratio between a ^12^C_2_-acetyl-peptide and the same peptide carrying a ^13^C_2_-acetyl-group was manually calculated as previously described [51].

### LC-MS/MS Analysis

The digests prepared above were analyzed by data-dependent LC-MS/MS with collision-induced dissociation (CID) using a Thermo Fisher Scientific Fusion Lumos mass spectrometry system [52]. The HPLC column was a Dionex 15 cm × 75 μm id Acclaim Pepmap C18, 2 μm, 10 nm reversed-phase capillary chromatography column. The peptides were eluted from the column by an acetonitrile/0.1% formic acid gradient at a flow rate of 0.3 μL/min.

### Statistical Analysis of Mass Spectrometry Lysine Modification Data

The mass spectrometry-derived peak ratios indicating the extent of chemical acetylation of lysines in the HPV16 capsid protein in the presence and absence of HD5 were analyzed with the Wilcoxon Signed Rank statistical test. This test is useful for analyzing matched paired data sets with non-parametric distributions. The one-tailed version of this test was used as the experimental ratios were compared to an expected value of 1, which would indicate no difference in lysine acetylation in the presence or absence of HD5. This test requires a minimum of 5 data points, therefore lysine acetylation data for 2 L1 residues, K59 and K479, was excluded from Table 2. The lysine acetylation data for one L2 residue, K318, was excluded from Table 3 as it was found to be not statistically significant.

### Structural Modeling

The UCSF Chimera [53] “Build Structure” and “Adjust Torsion” commands were used to build the starting conformation of an extended model for the C-terminal tail of 1 L1 subunit in PDB: 5KEP (chain F, modeled residues T481-L505). The backbone torsion angles of the modeled region were adjusted so that the C-terminal tail would pass through a capsid opening and extend away from the capsid. Minor backbone torsion angle adjustments were made with the Swiss-PDB Viewer Ramachandran Plot tool [54]. The C-terminal tail model was validated with the RAMPAGE Ramachandran plot analysis website [55] and found to have all residues in favored regions. The UCSF Chimera “Find Clashes/Contacts” command was used to check for clashes with the surrounding L1 chains.

To model the possible interactions between this extended model of the L1 C-terminal tail and an HD5 dimer, MD simulations were performed with NAMD [56] using implicit solvent and the CHARMM27 force field. Input coordinates were generated with 1 L1 Chain F (aa 8-505) and an HD5 dimer (PDB: 1ZMP) with 10 different starting positions for HD5 relative to the L1 C-terminal tail. MD simulations (5 nanoseconds) were performed, and nonbonded energies between the L1 Chain F and the HD5 dimer were calculated for the ending coordinates using the NAMD Energy plugin in VMD [57]. The MD simulation with the most favorable nonbonded interaction energy (-322 kcal/mol) at the end of the simulation was selected. This interaction energy is predominantly due to electrostatic interactions (-227 kcal/mol) with a smaller van der Waals component (-94 kcal/mol). The MD simulations were performed on the Case Western Reserve University High Performance Computing Cluster.

### Accession Numbers

CryoEM maps for the HPV16+HD5 complex and HPV16 have been deposited in the EM database (www.emdatabank.org/) with respective accession numbers EMD: 20218 and 20219.

## RESULTS

### Sub-Nanometer Resolution Structures of HPV16 and HPV16+HD5

**Figure 1.**
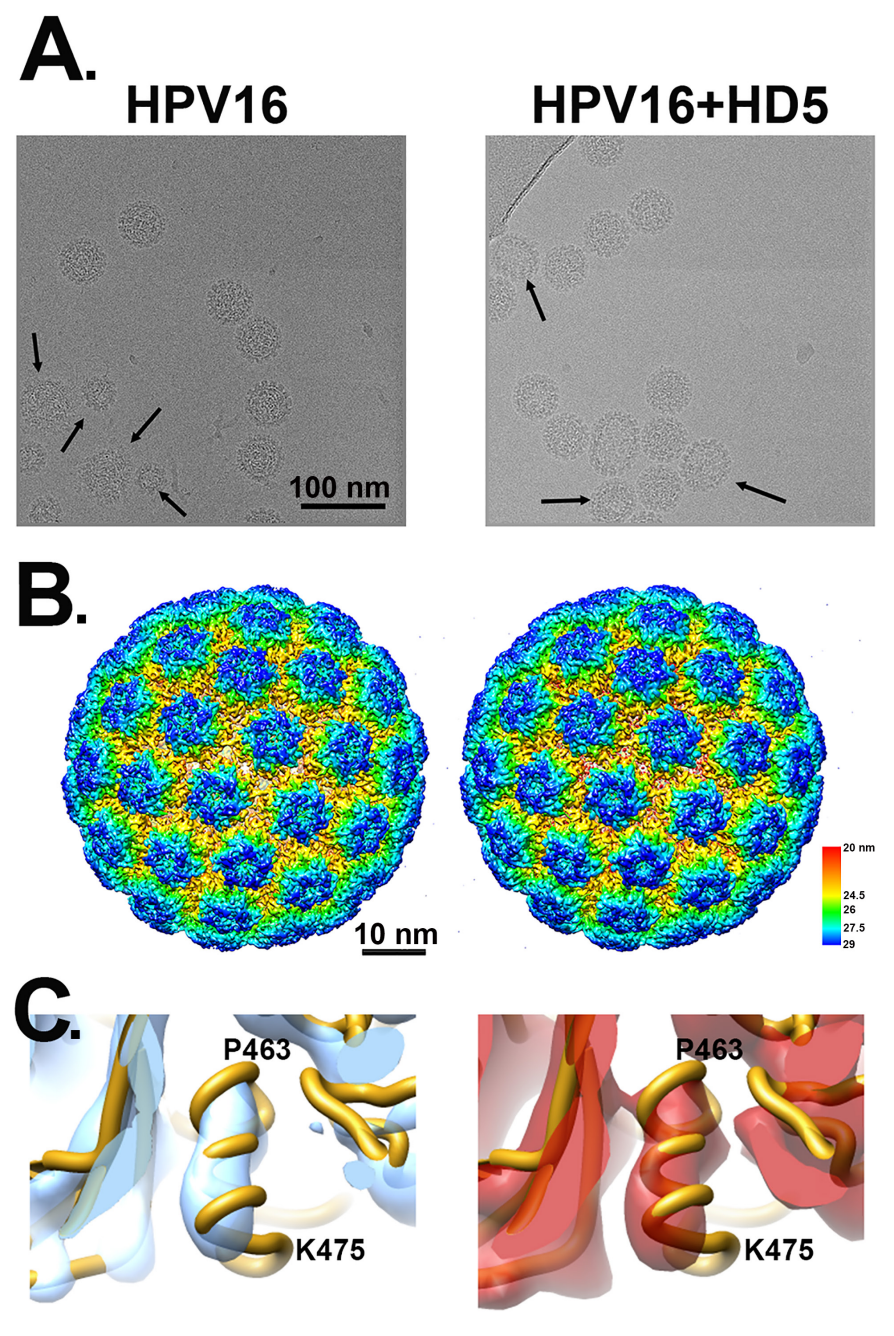
**CryoEM of HPV16 and HPV16+HD5.** (A) Representative cryo-electron micrographs of HPV16 (left) and HPV16+HD5 (right). Arrows point to capsids with heterogeneous size or shape. Scale bar = 100 nm. (B) Radially colored representations of HPV16 (left) and HPV16+HD5 (right). Scale bar = 10 nm. (C) Enlarged views of the HPV16 (left) and HPV16+HD5 (right) cryoEM maps showing α-helical density in L1 (aa 463-475, chain E) in both structures.

**Supplementary Figure 1.**
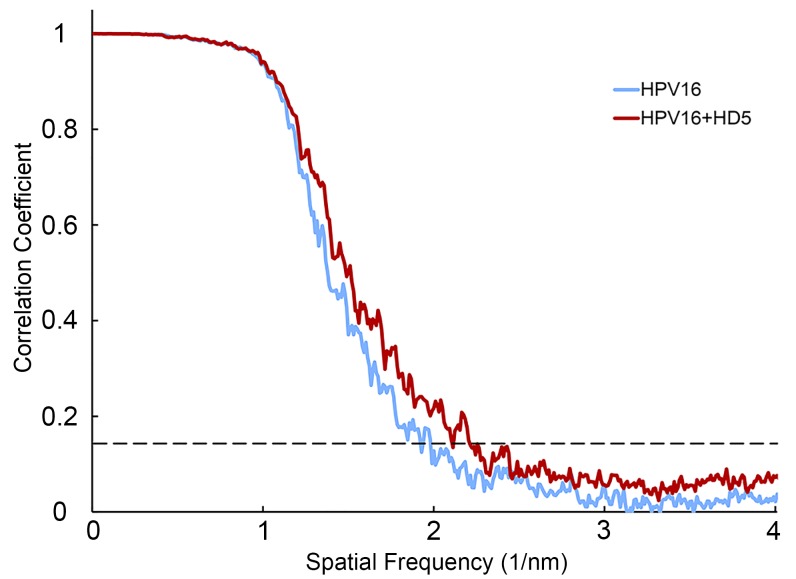
**Fourier Shell Correlation (FSC) plots for HPV16 and HPV16+HD5 cryo-EM reconstructions.** FSC plots calculated by RELION are shown with the HPV16 plot in blue and the HPV16+HD5 plot in red. The resolutions indicated by the 0.143 threshold (dashed line) are 0.53 nm for HPV16 and 0.49 nm for HPV16+HD5.

Samples of HPV16 pseudovirus (PsV) and HPV16 PsV in complex with a neutralizing concentration of HD5 (10µM) were vitrified, and cryoEM data collection was performed on a Titan Krios with a Direct Electron DE20 detector. Motion-corrected and dose-compensated micrographs of both samples showed well-dispersed particles with similar morphology: spherical capsids and prominent protrusions for the capsomers (Figure 1A). HPV16 PsVs have diameters of ~60 nm, consistent with previous structures [29, 47]. However, the viral capsids in the cryo-electron micrographs are not completely uniform, with heterogeneity in both size and shape (Figure 1A, arrows). Approximately 26% of particles in both the HPV16 and HPV16+HD5 datasets were observed to have either a non-round shape or a diameter smaller than 60 nm. The presence of HD5 did not affect the fraction of particles that were non-spherical. REgularized LIkelihood OptimizatioN (RELION) was used to sort the populations of HPV16 and HPV16+HD5 particles to include only those with spherical shape and a uniform diameter to produce a more homogenous dataset for reconstruction [59]. The reconstruction of HPV16 resulted in a 0.53 nm structure, while HPV16+HD5 resulted in a 0.49 nm structure (Figure 1B), as determined using gold standard Fourier shell correlation (FSC) curves at the 0.143 threshold (Figure S1). At this resolution, both HPV16 and HPV16+HD5 reveal α-helical features (Figure 1C) and have similar capsid structures. Subtraction of the HPV16 structure from the HPV16+HD5 structure did not result in difference density in the capsid that might be attributed to HD5 binding. Therefore, we hypothesized that HD5 binds to flexible or heterogeneous regions on the HPV16 capsid, which are not resolved.

### HD5 Stabilizes the Capsid/Core Interaction

Prior cryoEM studies of HPV16 at pseudoatomic resolution indicate that the N- and C-terminal regions of L1 are disordered [29, 47]. In the high resolution cryoEM study by Guan *et al*, the 6 L1 chains in the asymmetric unit are slightly different and are missing between 2 and 17 residues at the N-terminus and between 19 and 25 residues at the C-terminus. No density is observed for the L1 termini in either the HPV16 or HPV16+HD5 cryoEM structures presented here, indicating that they remain disordered in the presence of HD5. In addition, like the near-atomic resolution cryoEM structure of HPV16 by Guan *et al*, the HPV16 cryoEM structures presented here do not reveal any well-defined density for L2. While surface-exposed regions of L2 and disordered N-and C-terminal tails of L1 could be involved in HD5 binding, these interactions might not result in any obvious density within the HPV16+HD5 cryoEM structure.

**Figure 2.**
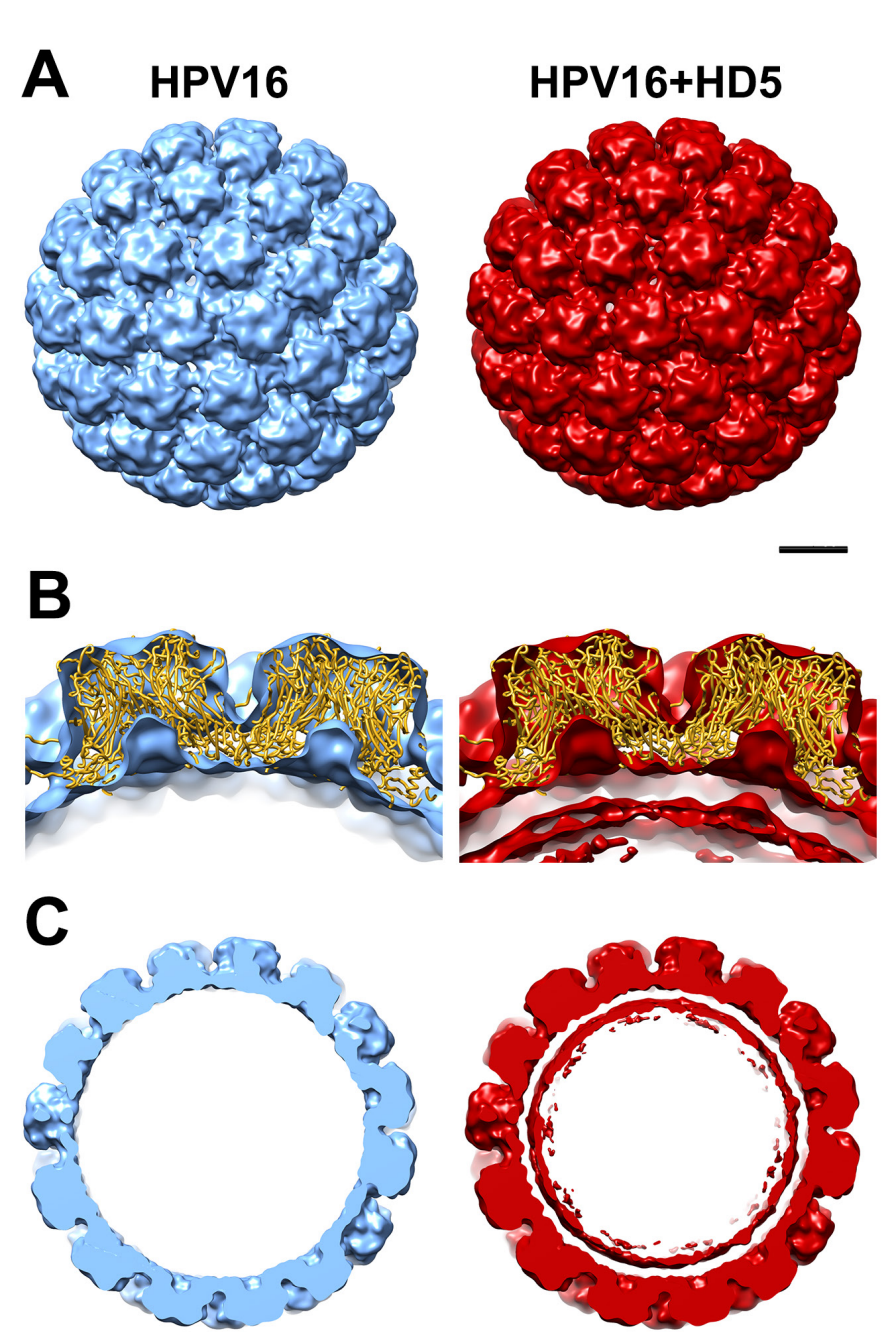
**Filtered maps of HPV16 and HPV16+HD5.** (A) Both cryoEM structures are shown filtered to 2 nm. Scale bar = 10 nm. (B) Cross-sectional views with docked L1 capsid coordinates (PDB: 5KEP) shown as a ribbon (gold) and oriented with the hexavalent L1 pentamer on the left and the pentavalent L1 pentamer on the right. In the HPV16+HD5 structure, additional density is observed below the L1 capsid. (C) Central slabs (10 nm thick) of both structures. Note the prominent internal core density below the L1 capsid in the HPV16+HD5 structure.

To investigate the possibility that HD5 binds to protein regions on the capsid that have a limited degree of flexibility, we filtered the cryoEM density map, resulting in lower resolution but a higher signal-to-noise ratio for regions of limited mobility. However, even filtering the HPV16 and HPV16+HD5 structures to 1, 1.5, and 2 nm still did not reveal any obvious density for HD5 on the capsid (Figure 2A). The cryoEM-derived L1 capsid coordinates fit well within both the HPV16 and HPV16+HD5 structures filtered to 2 nm resolution leaving no unexplained density that might be assigned to L2, the termini of L1, or HD5 (Figure 2B). However, we noted that the internal core density for HPV16+HD5 was different from that observed for HPV16 (Figure 2C). Thus, although there was no discernible change in capsid protein density, we did observe an effect of HD5 binding on the virion structure.

**Figure 3.**
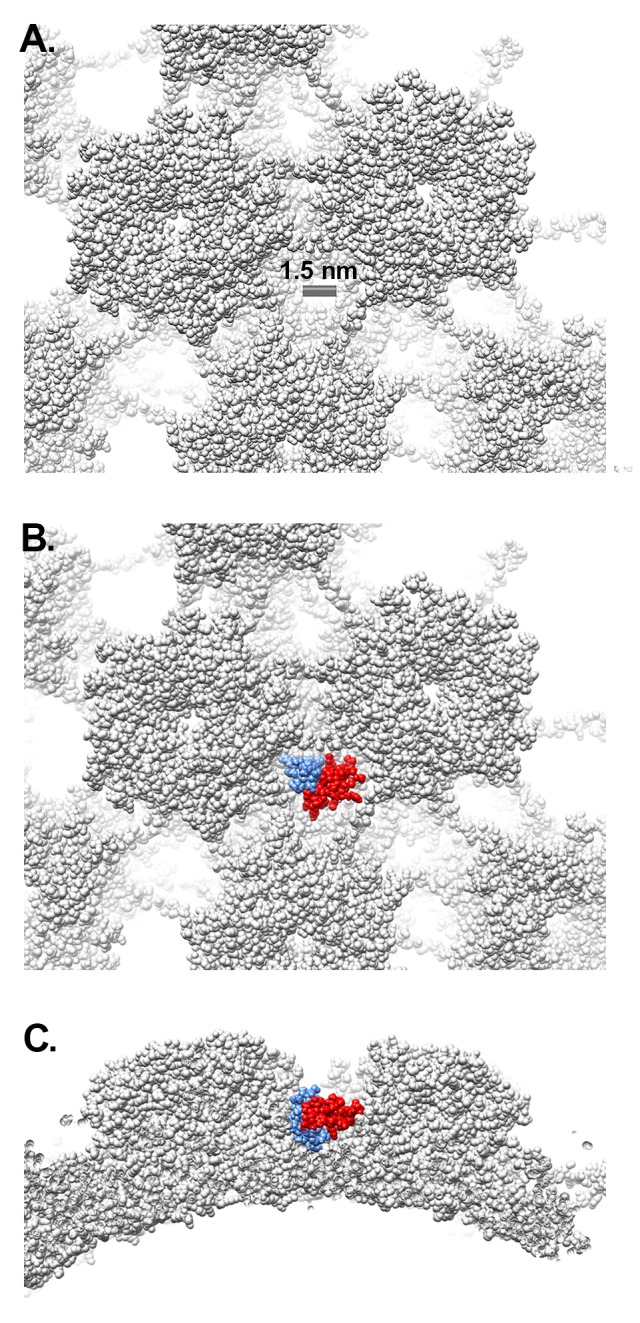
**Gaps in the L1 capsid are smaller than HD5.** (A) Space-filling representation of the L1 capsid with a scale bar indicating a capsid gap ~1.5 nm in diameter (PDB: 5KEP). (B) Space-filling representation of an HD5 dimer (red and blue) (PDB: 1ZMP, chains A and C) positioned within a depression between L1 pentamers. (C) Side view of panel B.

**Figure 4.**
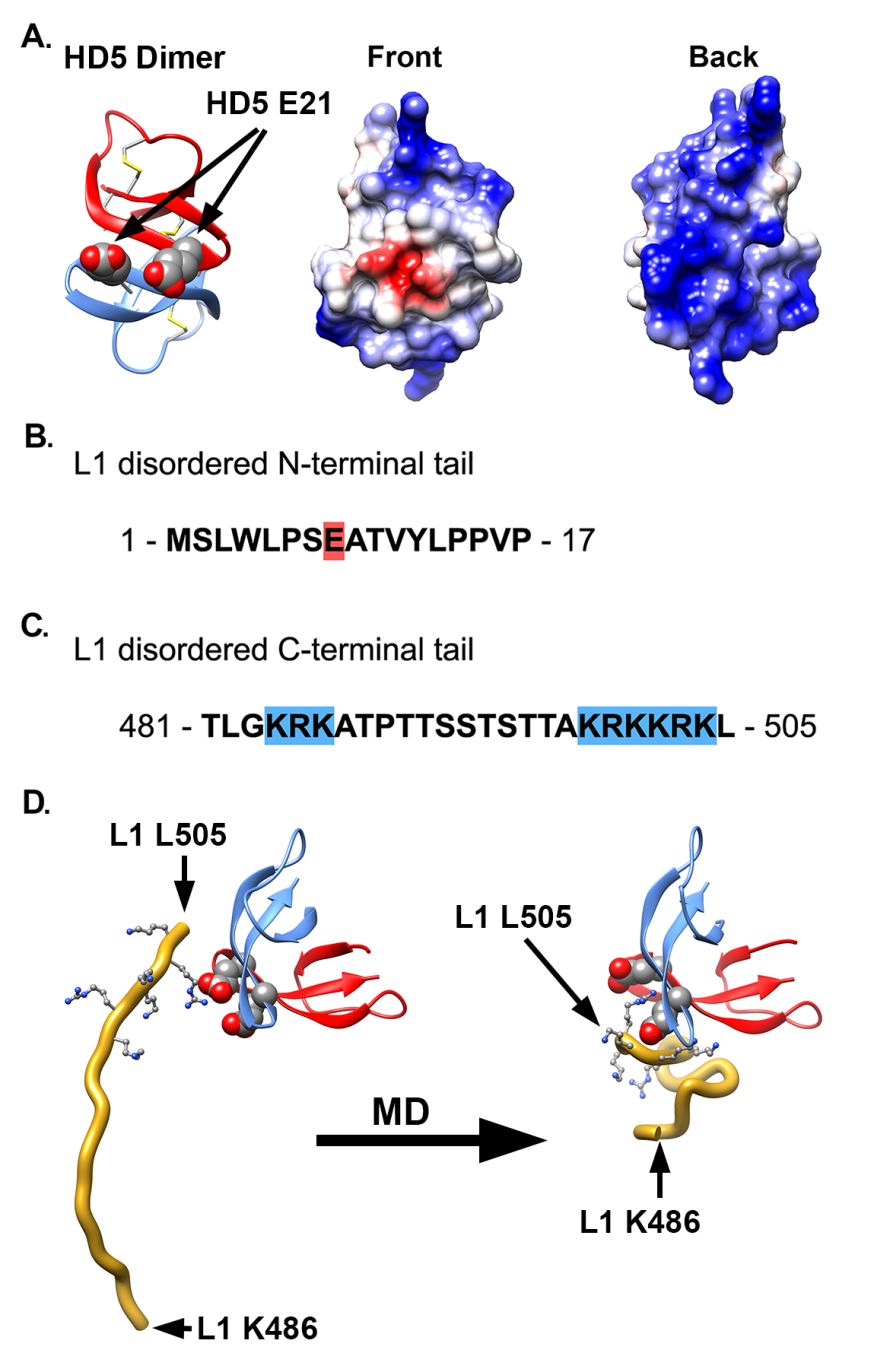
**Proposed interaction between HD5 dimer and L1 C-terminal tail.** (A) Ribbon and Coulombic surface representations of an HD5 dimer (PDB: 1ZMP, chains A and C). Disulfide bonds are depicted in stick representation and E21 residues in space-filling representation. The 2 E21 residues are next to each other in the dimer and form a negatively charged patch (red) on the Coulombic surface of a predominantly positively charged Coulombic surface. (B) Sequence of the longest disordered L1 N-terminal tail (aa 1-17) in the near-atomic resolution cryoEM-based HPV16 structure (PDB: 5KEP). (C) Sequence of the longest disordered L1 C-terminal tail (aa 481-505). In panels B and C charged residues are indicated with red (negative) or blue (positive) highlights. (D) MD-derived model of the interaction between an HD5 dimer and the L1 C-terminal tail (aa 486-505, gold ribbon). Both the starting and ending coordinates are shown. The negatively charged residues of the HD5 dimer (space-filling representation) form favorable electrostatic interactions with positively charged residues in the L1 C-terminal tail (499-KRKKRK-504, ball and stick representation).

The HPV core includes portions of L2 and the genome, or in the case of PsV, randomly incorporated DNA. There is no evidence to suggest that the core of HPV16 has a regular structure, and application of icosahedral symmetry during the reconstruction process leads to most of the core density being averaged away. Accordingly, little to no core density is observed in the near-atomic resolution cryoEM structure of HPV16 by Guan *et al* [29] or in the near-atomic resolution HPV16 cryoEM structure presented here. Thus, the increased core density in the filtered HPV16+HD5 structure is striking and suggests a specific effect of HD5. A simple explanation might be that HD5 has penetrated the capsid and is bound to flexible regions of L1 or L2 or to the DNA inside the capsid. Although there are small openings in the HPV16 capsid (up to ~1.5 nm in diameter, Figure 3A), even an HD5 monomer (dimensions ~2 × 2.5 × 3.5 nm) is too large to enter into the core of the virion. The HD5 monomer contains 3 intramolecular disulfide bonds (Figure 4A). A linear form of HD5 without disulfide bonds might be able to enter the interior of the virion, but the properly folded, disulfide-bonded form of HD5, as used in our HPV16 + HD5 cryoEM study, would likely be restricted to binding on the capsid exterior. Also, antiviral activity of HD5 against HPV16 requires dimerization [20], and the HD5 dimer (~2.5 × 4 × 4 nm) is even more unlikely to pass through the small openings in the HPV16 capsid. Therefore, we conclude that HD5 does not enter into the interior of the virion, that the effect of HD5 on the HPV16 core is indirect, and that HD5 binding occurs on the exterior surface of the capsid.

### Crosslinking Mass Spectrometry Indicates HD5 Binding Near L1 C-Terminal Tails

During HPV entry, the virus uncoats, resulting in separation of most of L1 from a complex of L2 and genome [60-62]. Cell culture studies indicate that a major mechanism for HD5 inhibition of HPV16 infection is to prevent this separation [19]. If HD5 binds to disordered and highly flexible regions of both L1 and L2 on the exterior of the capsid, this would effectively entangle the 2 proteins and cement interactions between disordered regions of L1 and L2, leading to reduced degrees of freedom for L2 and a tighter association between L1 and the core components (L2 and DNA).

**Supplementary Figure 2.**
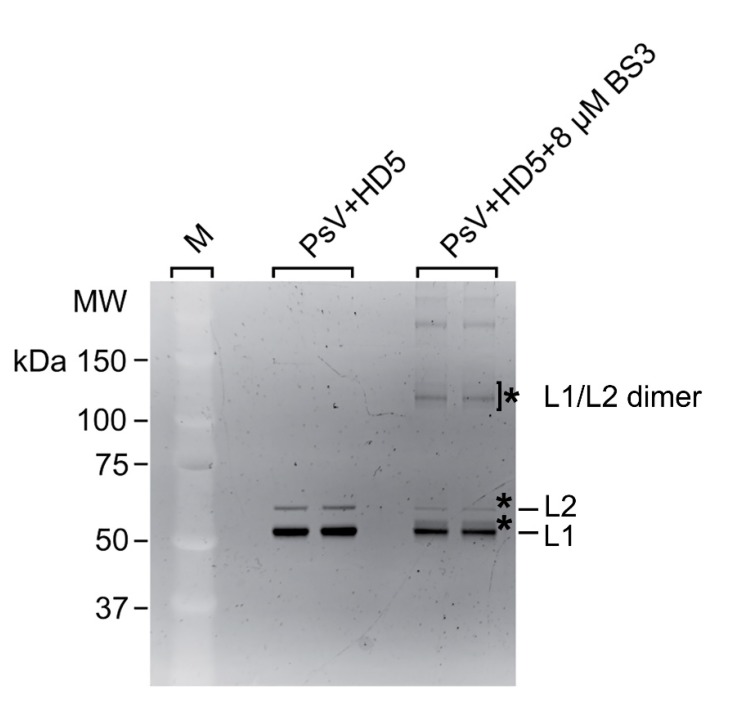
**SDS PAGE used in BS3 crosslinking mass spectrometry study.** Sample lanes with HPV16 pseudovirus (PsV) plus HD5 both with and without 8 μM BS3 are indicated along with a molecular weight marker lane (M). A mutant HD5 containing substitutions of two arginine residues (R13 and R32) for lysine (HD5-R13K/R32K) was used for this study to provide two additional primary amines for crosslinking. The gel was stained with Sypro Ruby, which is compatible with mass spectrometry. In the absence of BS3, clear bands are detected for L1 and L2. In the presence of BS3, L1 and L2 bands are detected, along with a dispersed L1/L2 dimer band and higher bands indicating multimeric crosslinking by BS3. In addition, a faint band is detected above the L1 band. Three gel regions, as indicated by asterisks, were excised for the BS3 crosslinking mass spectrometry study: 1) the faint band above the L1 band, 2) a presumed (nonvisible) band above the L2 band, and 3) the dispersed L1/L2 dimer region.

**Supplementary Figure 3.**
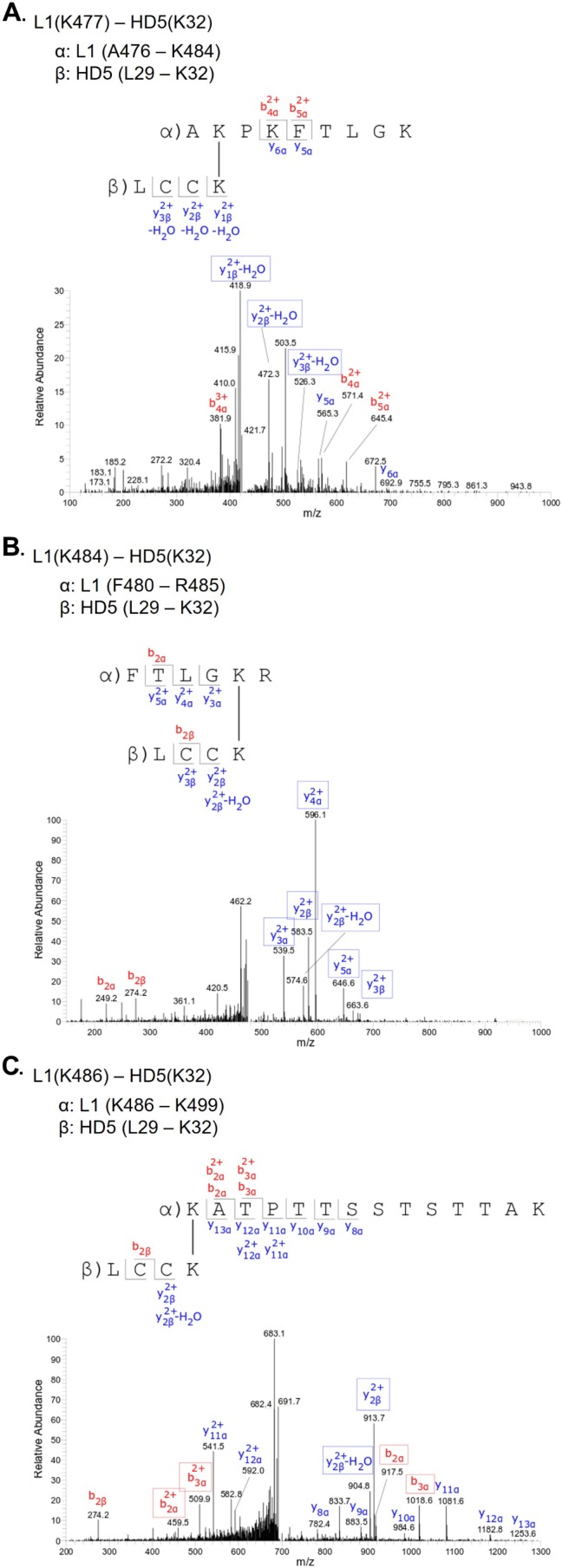
**Tandem mass spectra of crosslinked peptides identified: Inter-protein cross-links between L1 and HD5-R13K/R32K.** Observed b- and y-series ions are shown in each spectrum and the fragment ion species containing the crosslinker plus regions of two crosslinked peptides are boxed. (A) MS/MS spectrum of an inter-protein crosslinked peptide (m/z 427.7, +4 charge) between Lys477 of L1 (Ala476 – Lys484) and Lys32 of HD5 (Leu29 – Lys32); precursor ion = m/z 427.7. (B) MS/MS spectrum of an inter-protein crosslinked peptide (m/z 480.3, +3 charge) between Lys484 of L1 (Phe480 – Arg485) and Lys32 of HD5 (Leu29 – Lys32). (C) MS/MS spectrum of an inter-protein crosslinked peptide (m/z 700.3, +3 charge) between Lys486 of L1 (Lys486 – Lys499) and Lys32 of HD5 (Leu29 – Lys32).

**Supplementary Figure 4.**
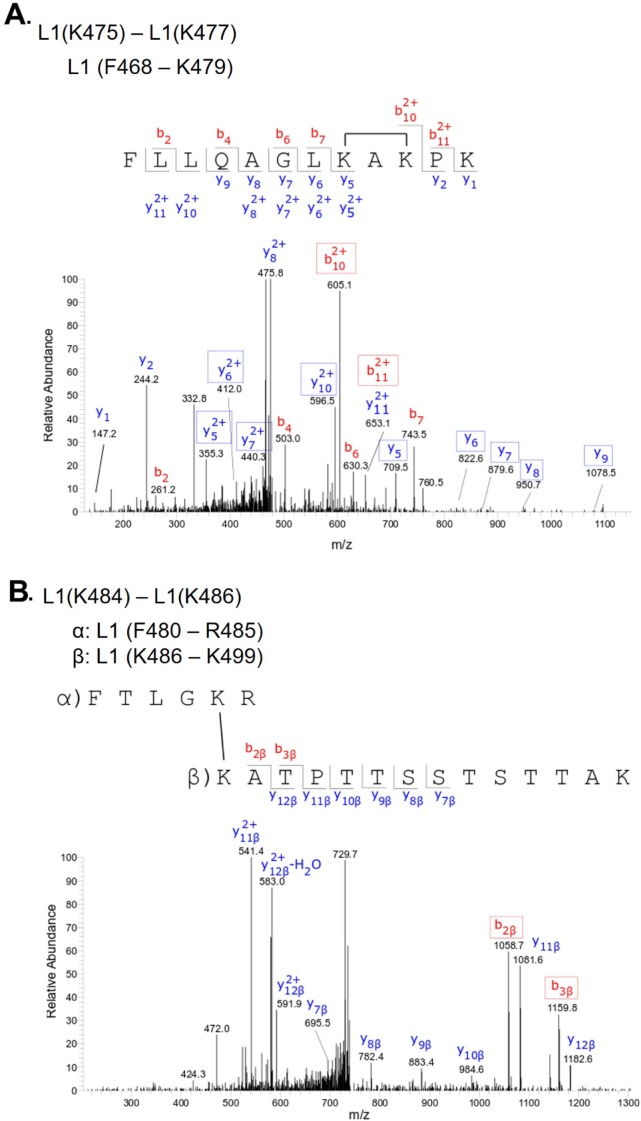
**Tandem mass spectra of crosslinked peptides identified: Intra-protein cross-links within L1.** Observed b- and y-series ions are shown in each spectrum and the fragment ion species containing the crosslinker plus regions of two crosslinked peptides are boxed. (A) MS/MS spectrum of a crosslinked peptide (m/z 484.6, +3 charge) between Lys475 and Lys477 of L1 (Phe468 – Lys479). (B) MS/MS spectrum of a crosslinked peptide (m/z 747.4, +3 charge) between Lys484 and Lys486 of L1 (Phe480 – Arg485 and Lys486 – Lys499).

**Supplementary Figure 5.**
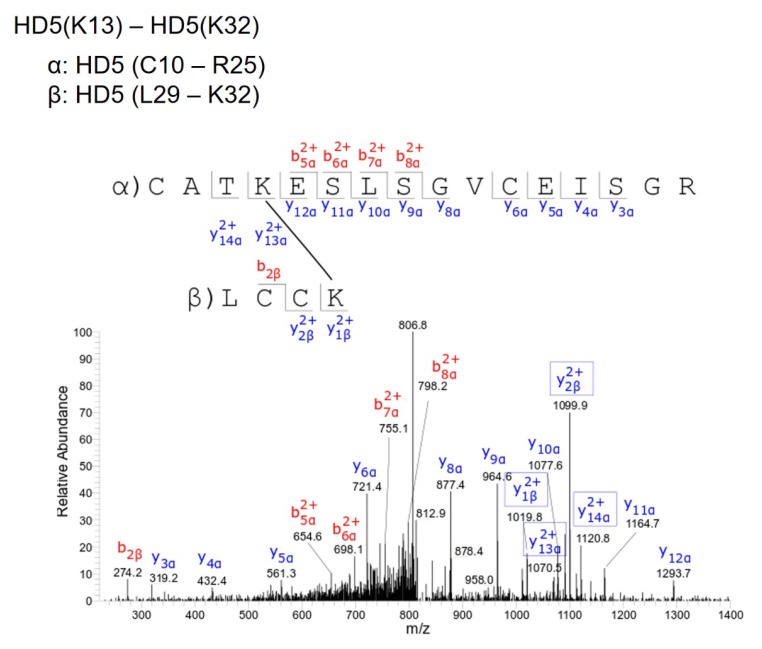
**Tandem mass spectra of crosslinked peptides identified: Intra-protein crosslinks within HD5-R13K/R32K.** Observed b- and y-series ions are shown in each spectrum and the fragment ion species containing the crosslinker plus regions of two crosslinked peptides are boxed. MS/MS spectrum of a crosslinked peptide (m/z 824.4, +3 charge) between Lys13 and Lys32 of HD5 (Cys10 – Arg25 and Leu29 – Lys32).

To investigate this mechanism, we performed crosslinking studies with BS3 to identify lysine residues in the vicinity of HD5 binding sites on the HPV16 capsid. For these studies, we used a mutant HD5 containing substitutions of 2 arginine residues (R13 and R32) for lysine (HD5-R13K/R32K), thereby introducing 2 additional primary amines for crosslinking. The HD5-R13K/R32K analog retains near wild-type antiviral activity against HPV16 PsV, unlike other HD5 analogs tested [17, 20]. Crystal structures of multiple HD5 analogs have been determined to have the same fold as wild-type HD5 [63-66], and the mass and HPLC retention time of the HD5-R13K/R32K analog during production and purification indicated that its fold is equivalent to wild-type HD5. HPV16 PsV was incubated with 20µM HD5-R13K/R32K to allow binding. The complex was incubated with 8µM BS3 followed by SDS-PAGE, resulting in the formation of multiple species with altered mobility compared to control (Figure S2). Three regions were excised from the SDS-PAGE gel, digested by trypsin, and analyzed by data-dependent LC-MS/MS with collision-induced fragmentation (CID) (Figures S3, S4, and S5). MS/MS analysis confirmed inter-protein crosslinks between L1 and HD5-R13K/R32K (Table 1), as well as intra-protein crosslinks within L1 (Table S1) and within HD5-R13K/R32K (Table S2). Because HD5-R13K/R32K has near wild-type antiviral activity, and the 2 substitutions of arginine to lysine are relatively conservative, the MS/MS finding of crosslinks between HD5-R13K/R32K and the L1 C-terminal tails should also be relevant for wild-type HD5. The observed crosslinks between L1 and HD5 all involve L1 residues near the C-terminus, K477, K484, and K486.

**Table 1.** Mass spectrometry detected BS3 crosslinks between L1 and HD5-R13K/R32K

BS3 crosslinked residues	Band position on gel
L1(K477) - HD5(K32)	Above L1 band
L1(K484) - HD5(K32)	Above L1 band
L1(K486) - HD5(K32)	L1/L2 dimer region

### Differential Lysine Modification Mass Spectrometry Indicates Regions of L1 and L2 Affected by HD5

An additional mass spectrometry experiment was conducted to assess which regions of the HPV16 capsid are shielded by HD5 from chemical lysine acetylation. Unlabeled and ^13^C-labeled acetic anhydride samples were used to modify either HPV16 PsV alone or HPV16 PsV+HD5. Both forward (^13^C-labeled acetic anhydride with HPV16 PsV+HD5) and reverse (^13^C-labeled acetic anhydride with HPV16 PsV) experiments were conducted. The ratios of the ^12^C- and ^13^C-labeled mass spectrometry peaks were analyzed. A ratio equal to 1 would indicate no difference in lysine acetylation in the presence or absence of HD5. A ratio less than 1 would indicate that HD5 reduces acetylation at that site, while a ratio of greater than 1 would indicate that HD5 enhances acetylation at that site. Acetylation of 8 lysines in L1 and 2 lysines in L2 was found to be affected by the presence of HD5 (Tables 2 and 3). L1 lysine residues K430, K437, K442, K475, K484, K486, and K499, and L2 lysine residues K20 and K309, were all shielded by HD5 from chemical acetylation. For the remaining lysine, L1 K467, HD5 had the opposite effect and enhanced acetylation.

**Table 2.** Mass spectrometry detected lysine acetylation sites of L1 protein affected by the presence of HD5

Lysine Residue	Mean Ratio (+HD5/-HD5)	Standard Deviation	Number of data points (n)	*P*-value	Effect of HD5
K430	0.36	0.05	5	0.05	HD5 reduces acetylation
K437	0.78	0.10	6	0.025	HD5 reduces acetylation
K442	0.66	0.17	6	0.025	HD5 reduces acetylation
K467	1.25	0.12	6	0.025	HD5 ***enhances*** acetylation
K475	0.72	0.06	6	0.025	HD5 reduces acetylation
K484	0.62	0.18	6	0.025	HD5 reduces acetylation
K486	0.71	0.24	6	0.025	HD5 reduces acetylation
K499	0.73	0.24	6	0.025	HD5 reduces acetylation

**Table 3.** Mass spectrometry detected lysine acetylation sites of L2 protein affected by the presence of HD5

Lysine Residue	*P*-value	Mean Ratio (+HD5/-HD5)	Standard Deviation	Number of data points (n)	Effect of HD5
K20	0.25	0.26	5	0.05	HD5 reduces acetylation
K309	0.75	0.24	6	0.025	HD5 reduces acetylation

The simplest interpretation of the acetylation results for most of the L1 residues is that HD5 bound to the HPV16 capsid sterically blocks access of acetic anhydride to these sites, which is supported by the absence of HD5-induced conformational changes in the ordered region of L1. In contrast, the mass spectrometry results indicate that L1 K467 becomes more accessible to acetic anhydride after HD5 is bound to the virion, suggesting greater solvent accessibility for that particular residue in the presence of HD5. Since we observed no conformational changes in the ordered portion of the L1 capsid due to HD5 binding, the finding of enhanced acetylation at L1 K467 suggests a change in either L2 or the disordered termini of L1. Moderate resolution cryoEM reconstructions indicate that a portion of L2 is arranged within internal cavities of the L1 pentamers [40], and residue K467 is located in this vicinity within the internal cavity of the L1 pen-tamer. Thus, a shifting of L2 within the virion when HD5 binds could lead to greater exposure of L1 K467.

The differential lysine acetylation results also indicate that the presence of HD5 affects 2 lysine residues in L2 (Table 3). Both L2 K20 and L2 K309 show reduced acetylation in the presence of HD5. During cell entry, 12 residues at the N-terminus of HPV16 L2 are removed by cleavage by the host protease furin [67]. Thus, the N-terminal tail of L2 is at least transiently exposed on the virion surface [41, 43, 68-72]. Protrusion of the N-terminal tail of L2 from a capsid opening near an HD5 binding site could explain why the presence of HD5 causes a comparatively strong (4-fold) reduction in acetylation of L2 K20 and would also be consistent with HD5-mediated inhibition of L2 cleavage by furin [18]. Multiple regions of L2, in addition to the N-terminal tail, emerge from the virion and become accessible on the capsid surface [42, 73]. Exposure of a middle segment of L2 through a capsid opening near an HD5 binding site could explain why the presence of HD5 causes a reduction in acetylation of L2 K309.

### Proposed Interaction Between an HD5 Dimer and the L1 C-Terminal Tail

Based on the mass spectrometry results, we considered possible interactions between L1 and HD5. Examination of the crystal structure of an HD5 dimer (PDB: 1ZMP) indicates that the surface is mostly positively charged, with just 2 negatively charged residues (E21 from each monomer) forming a small negatively charged patch (Figure 4A) [74]. We previously identified E21 as one of the critical residues in HD5 for antiviral activity against HPV16 [17]. In addition, 2 additional critical residues, Y27 and L29, are at the HD5 dimer interface and may be important for maintaining the dimeric form of HD5. On the virus, the disordered N-terminal tail of L1 has only a single negatively charged residue (Figure 4B), while the disordered C-terminal tail has 2 regions of multiple positively charged residues (Figure 4C). The first ordered residues at the N-terminal end and the last ordered residues at the C-terminal end are positioned on the inner surface of the L1 capsid [29]. Potentially, both the disordered N- and C-terminal tails could protrude through gaps in the L1 capsid (Figure 3A). However, given that the C-terminal tail is both more hydro-philic and longer than the N-terminal tail, it seems more likely that the C-terminal tail is exposed on the exterior of the capsid. While exposure of the C-terminal tail may only be transient, it would still offer a potential binding site for HD5. Accordingly, we hypothesize that the most distal, positively charged region of the L1 C-terminal tail (499-KRKKRK-504) interacts with the negatively charged patch of an HD5 dimer formed by 2 copies of E21. The MD simulations with L1 and an HD5 dimer indicate that favorable electrostatic interactions can be formed (Figure 4D). Furthermore, modeling studies indicate that the disordered L1 C-terminal tail is long enough to protrude through openings in the HPV16 capsid. Interaction of HD5 dimers with L1 C-terminal tails protruding from the capsid would effectively tether HD5 within depressions between L1 pentamers of the HPV capsid (Figure 3B and C). Given the stretch of 6 positively charged residues near the L1 C-terminus, there could be multiple, electrostatically favorable, interactions formed between an HD5 dimer and L1. Both the disordered nature of the L1 C-terminal tails and the possibility of multiple distinct binding interactions between HD5 and L1 would make it likely that no cryoEM density would be observed for the L1 C-terminal tails and bound HD5 dimers within the capsid depressions in the HPV16+HD5 cryoEM structure. Therefore, although unproven, our models supported by MD simulations provide a plausible explanation for both the cryoEM and mass spectrometry results and a putative position for HD5 on the viral capsid.

### Structural Models Illustrating BS3 Crosslinks Between L1 and HD5

**Figure 5.**
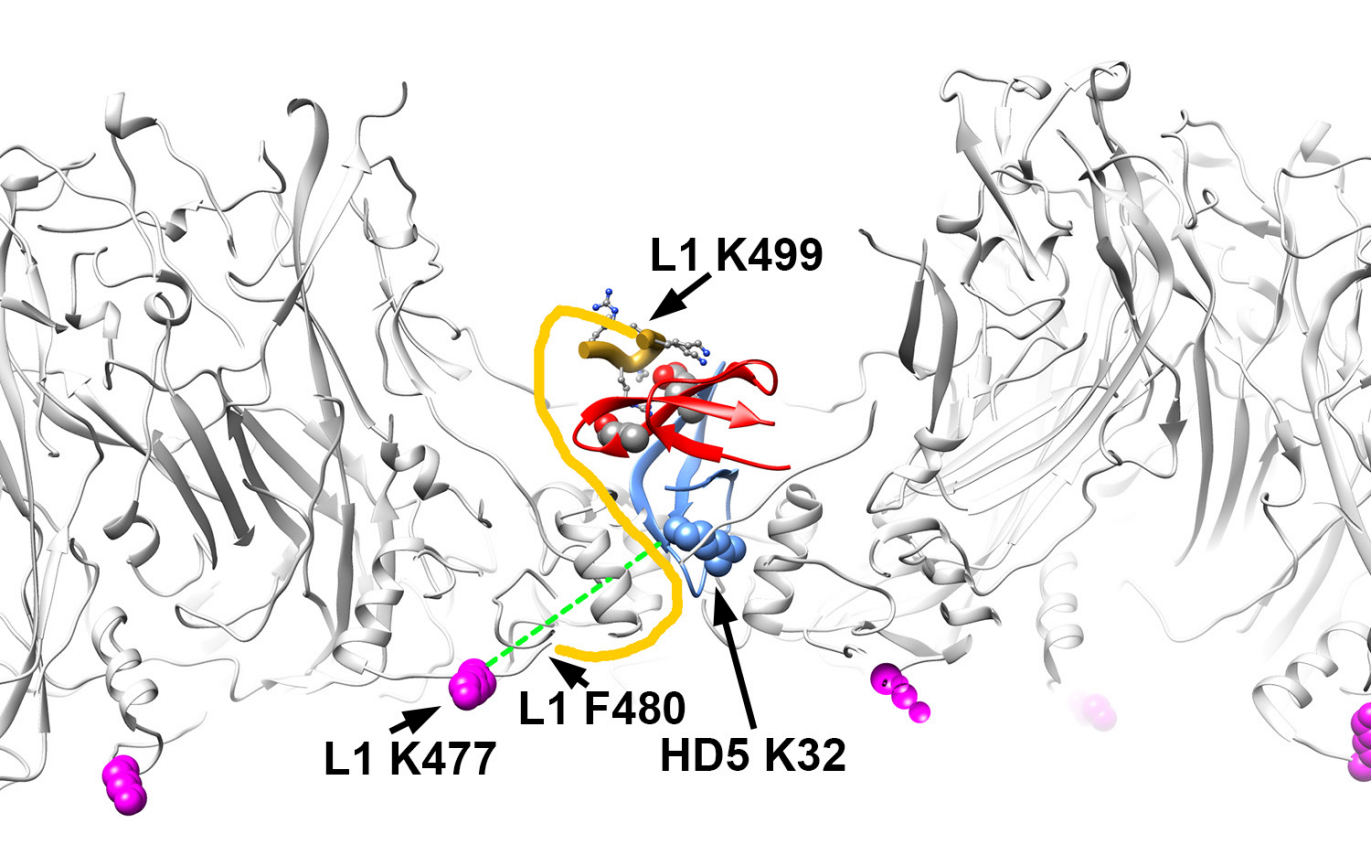
**Structural model illustrating BS3 crosslinks between L1 and HD5 lysine residues.** Side view of the L1 capsid (gray) with an HD5 dimer (red and blue) positioned within a depression between L1 pentamers such that one HD5 K32 residue (blue, space-filling) is directly above a capsid opening. The MD-derived model of an HD5 dimer and the last 6 residues of the L1 C-terminal tail (aa 499-505, gold ribbon) are shown as in Figure 4D. The flexible connecting residues between L1 F480 (Chain F) on the inner capsid surface and L1 K499 near the HD5 interaction site are depicted as a yellow line. The distance that would have to be spanned to connect these 2 residues (F480 and K499) is 3.7 nm, which is considerably less than the extended length of a 19 aa peptide (6.9 nm). Residue L1 K477 (magenta, space-filling) is detected as crosslinked to HD5 and is on the inner capsid surface. The dashed green line represents a distance of 2.8 nm and serves to indicate that the maximum distance constraint (3 nm) between Cα atoms of BS3 crosslinked lysines is met by this structural model.

We then determined whether positioning HD5 within depressions between L1 pentamers is consistent with our crosslinking mass spectrometry (CX-MS) results. We assumed a maximum distance of 3 nm between Cα atoms of BS3 crosslinked lysine residues [58]. We also noted that crosslinks only form on residues that are solvent accessible [75]. The CX-MS results indicate that a lysine on the inner capsid surface, L1 K477, is crosslinked to HD5. Since even an HD5 monomer is too large to pass freely through openings in the L1 capsid (Figure 3), we hypothesize that the chemical crosslinker must span the L1 capsid through a gap in the capsid. The cryoEM density of HPV16+HD5 indicates that the α-helix near K475 is still intact in the presence of HD5 (Figure 1C), suggesting no conformational change for this region of L1 in the presence of HD5. Positioning an HD5 dimer with one K32 residue directly above an opening in the L1 capsid places its Cα atom within ~2.8 nm of the Cα atom of the nearest L1 K477 on the inner capsid surface (Figure 5). The rest of the disordered L1 tail (yellow line in Figure 5) is sufficiently long (19 aa with a maximum extended length of 6.9 nm) to span the distance (3.7 nm) between the last ordered residue in the structure (F480) and the first residue supported by MD simulations to be in contact with HD5 (K499 in Figure 4D). Thus, this modeled HD5 position is feasible.

CX-MS indicates additional crosslinks between L1 (residues K484 and K486) and HD5 (Table 1). Since these 2 L1 residues (K484 and K486) are within the flexible connecting peptide between L1 F480 on the inner capsid surface and L1 K499 near the HD5 interaction site, it is difficult to measure the distance that would be spanned by a crosslinking molecule. For K484 and K486 to be within ~3 nm of HD5, the flexible L1 C-terminal tail likely bends up towards the surface of the virion, as we have modeled in Figure 5. Thus, with an HD5 dimer positioned within a depression between L1 pentamers and the L1 C-terminal tail protruding through a gap in the capsid, all of the cross-linked residues meet the spatial constraints of the experimental results.

### Structural Models Illustrating the Effect of HD5 on Acetylation of L1 and L2

**Figure 6.**
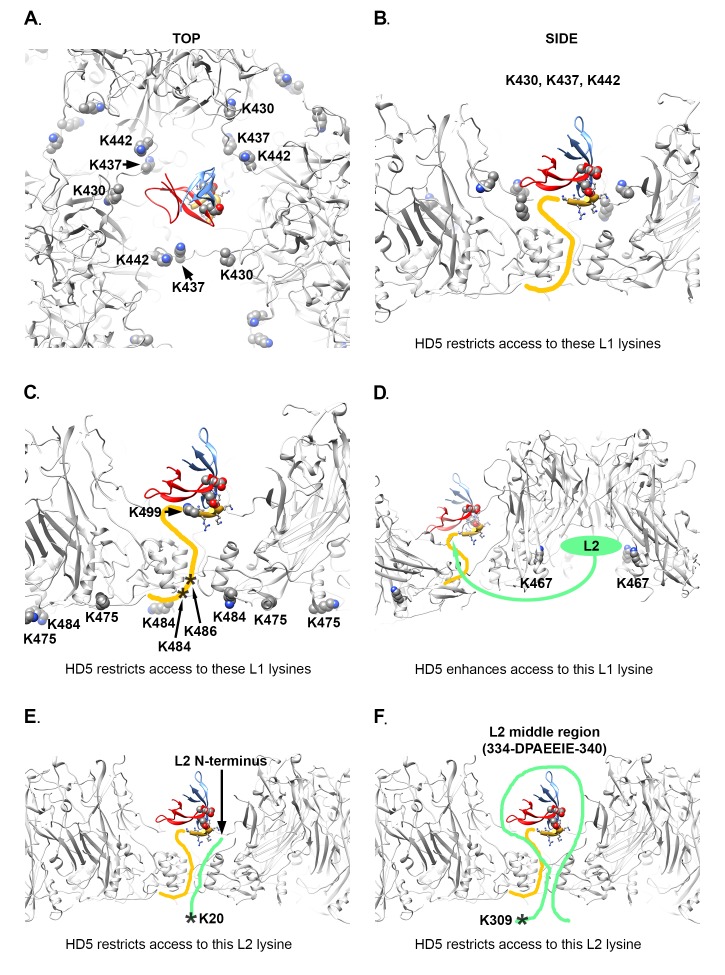
**Structural models illustrating effect of HD5 on acetylation of L1 and L2 lysine residues.** (A,B) Top and side views of the L1 capsid (gray) with the MD-derived model of an HD5 dimer and the last 6 residues of the L1 C-terminal tail (aa 499-505, gold ribbon) as in Figure 4D. Note that the MD-derived model is in a different orientation with respect to the HPV capsid compared to Figure 5. L1 residues K430, K437 and K442 (space-filling) from 3 L1 subunits surround the HD5 dimer in a capsid depression. (C) Same structural model with L1 residues K475 and K484 (space-filling) indicated on the inner capsid surface near a capsid gap and below an HD5 dimer. The approximate positions of L1 residues K484 and K486 are shown as asterisks for one flexible L1 C-terminal tail (yellow line). An additional L1 lysine (K499, space-filling) is shown as part of the MD-derived model of the HD5/L1 interaction. (D) Side and cropped view of the L1 capsid with 2 copies of residue K467 indicated within the internal cavity of an L1 pentamer. In this structural model, K467 residues are shielded from acetylation in the absence of HD5 by a disordered region of L2 binding within the L1 cavity. In the presence of HD5, a portion of L2 could be drawn toward HD5 (green arrow) thus leading to enhanced acetylation of K467. (E) Side view of the L1 capsid with an extended model for one L2 N-terminal tail (green) represented to indicate that L2 residue K20 (asterisk) could be located near a capsid gap and below an HD5 dimer. (F) Side view of the L1 capsid with an extended model for a middle portion of L2 (green) represented to indicate that L2 residue K309 (asterisk) could be located near a capsid gap and below an HD5 dimer. In this model the most negatively charged region of L2 (334-DPAEEIE-340) is drawn toward the HD5 dimer. The MD-derived model is in the same orientation with respect to the HPV capsid in panels A-F.

The mass spectrometry acetylation data is also consistent with the structural model derived from the CX-MS and cryoEM results and MD simulations with an HD5 dimer positioned within a depression between L1 pentamers. Figure 6A, B, and C illustrate how an HD5 dimer bound to a protruding L1 C-terminal tail would restrict access to L1 residues K430, K437, K442, K475, and K484 in the HPV16 capsid. Two additional L1 residues with reduced acetylation in the presence of HD5 (K486 and K499) are within the flexible L1 C-terminal tail.

**Supplementary Figure 6.**
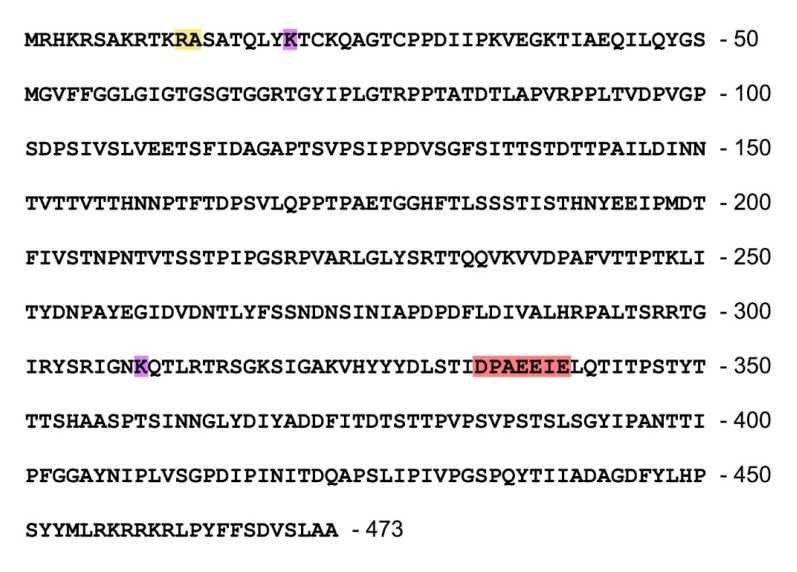
**HPV16 L2 sequence.** The most negatively charged region of the sequence is highlighted in red. The site of furin cleavage (between R12 and A13) (1), which is thought to be transiently exposed on the surface of the virion, is highlighted in yellow. The two lysines (K20 and K309) indicated by our mass spectrometry study to have restricted lysine modification in the presence of HD5 are highlighted in purple.

There is 1 L1 residue (K467) which displays enhanced acetylation in the presence of HD5. As mentioned above, residue K467 is located within the internal cavity of an L1 pentamer near an observed L2 binding site [40]. We propose that binding of HD5 to the HPV16 capsid induces a rearrangement of L2 within the virion which leads to increased solvent accessibility for L1 K467 (Figure 6D). We note that there is a negatively charged region within the HPV16 L2 sequence (Figure S6). Given that the HD5 dimer is mostly positively charged (Figure 4A), it seems plausible that the negatively charged region of L2 might shift its location within the virion after HD5 is bound.

Although there is no high-resolution structural information for L2, it is known that the L2 N-terminal region and various additional segments in the middle of L2 are transiently exposed on the surface of the virion [41-43, 68-73]. Even if L2 K20 remains inside the capsid, the presence of HD5 above the closest capsid opening could reduce access of acetic anhydride to this site (Figure 6E). In the case of L2 K309, this lysine residue is close to the negatively charged region (334-DPAEEIE-340) within the HPV16 L2 sequence (Figure S6). If the negatively charged region of L2 shifts its location within the capsid after HD5 is bound, this could plausibly block access of acetic anhydride to L2 K309 (Figure 6F).

The MD-derived model of the HD5 dimer and the C-terminal 6 residues of L1 is shown in the same orientation with respect to the HPV capsid in all panels of Figure 6, however this is a different orientation than shown in Figure 5. In Figure 6 the distance that would have to be spanned to connect L1 F480 on the inner capsid and L1 K499 in the MD-derived model is 3.2 nm, which is less than that in Figure 5, illustrating BS3 crosslinks between L1 and HD5 lysine residues. It is highly plausible that BS3 crosslinking would affect the position of the flexible L1 C-terminal tail and the bound HD5 dimer.

**Figure 7.**
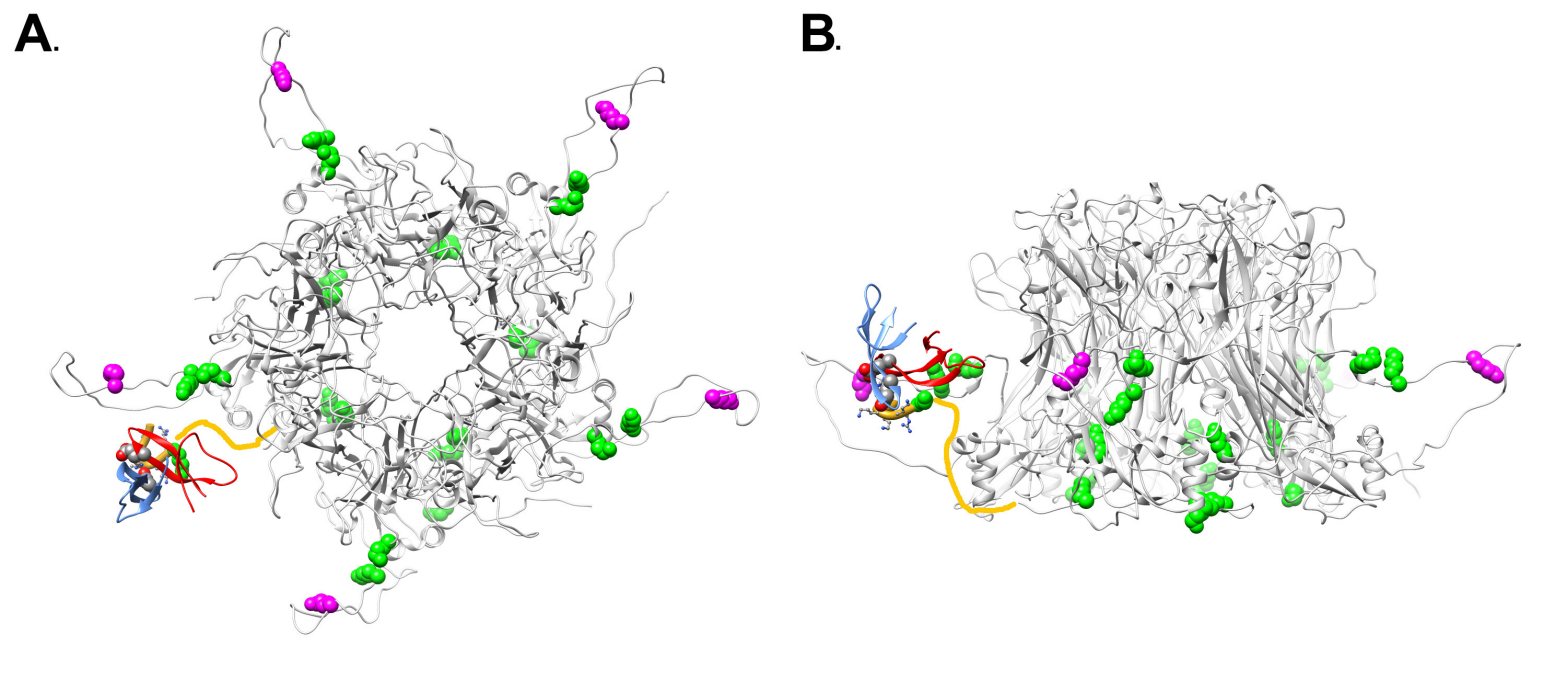
**Overview of L1 lysine residues affected by HD5 with modeled position of HD5 dimer and L1 C-terminal tail.** (A) Top view of an L1 pentamer (PDB: 5KEP, Chains B-F) with the lysine acetylation sites affected by the presence of HD5 in space-filling representation. The most affected lysine (K430) is in magenta and the other affected lysines (K437, K442, K467, K475, K484, K499) are in green. Some of the affected lysines are in the portion of the L1 C-terminal tail depicted as a yellow line (K484, K486). The MD-derived model of the HD5 dimer and the last 6 residues of the L1 C-terminal tail (aa 499-505) are in the same orientation with respect to the HPV capsid as in Figure 6. (B) Side view of panel A.

**Figure 8.**
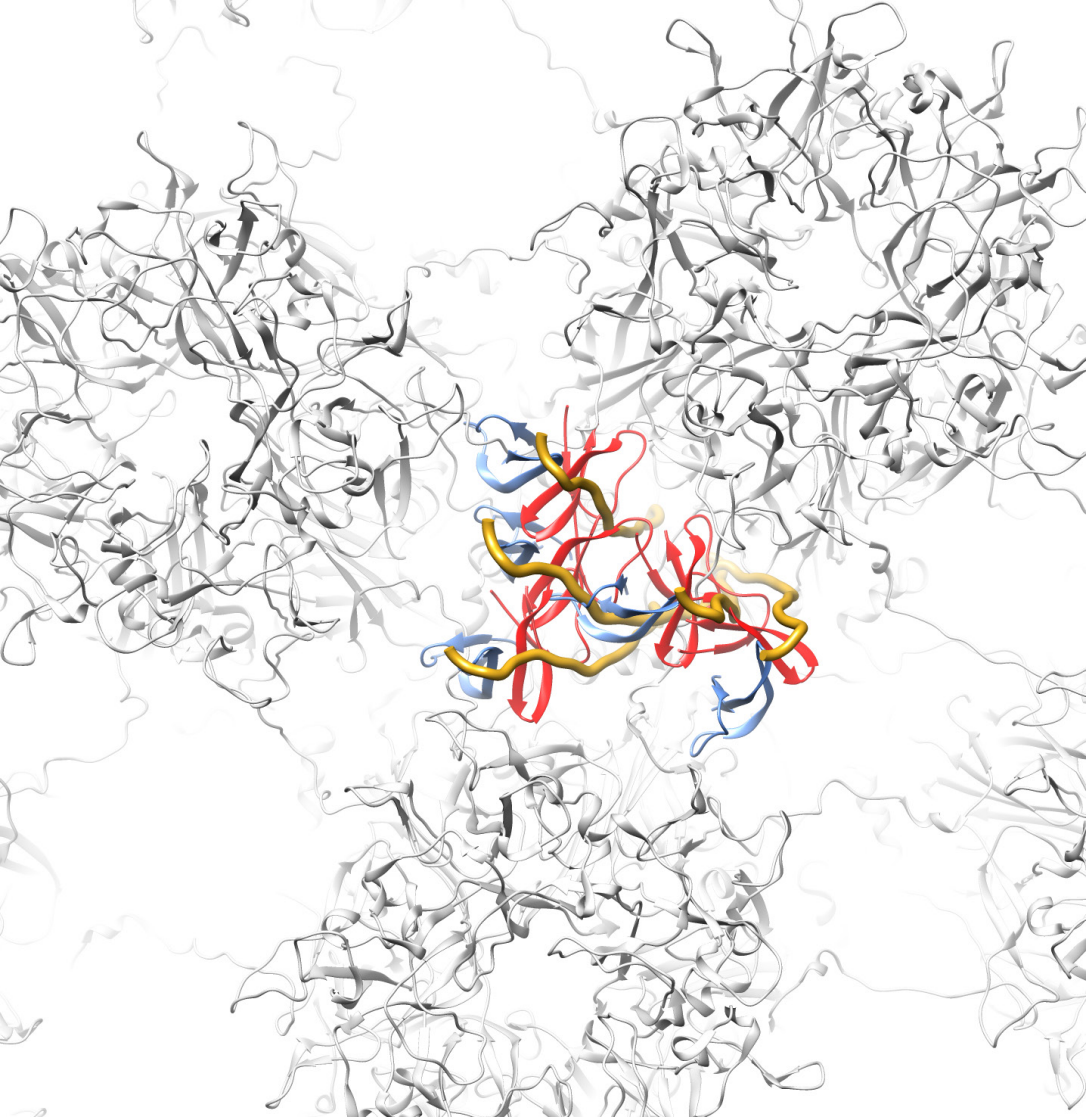
**Capsid depression with various possible positions for an HD5 dimer tethered by an L1 C-terminal tail.** Top view of a capsid depression between 3 L1 pentamers (PDB: 5KEP) with 5 overlapping copies of an extended model of the L1 C-terminal tail (aa 486-505, gold ribbon) an HD5 dimer (red, blue).

An overview of the differential lysine modification data for an L1 pentamer is shown in Figure 7, with the most highly affected lysine residue, K430, in magenta and the other affected lysine residues in green. It is notable that all of the affected lysines in the ordered portion of L1 are on the lower half of an L1 pentamer (Figure 7B). The proposed model for the HD5/HPV16 interaction places the most highly affected L1 residue (K430) in close proximity to HD5 (Figure 7A and B). The cryoEM structures of HPV16 and HPV16+HD5, the CX-MS data, and mass spectrometry acetylation data presented here are all in agreement with the proposed structural model with HD5 binding within a capsid depression and tethered to this site by a protruding disordered peptide of L1 and/or L2. It is important to note that tethering of an HD5 dimer to the HPV16 capsid via a disordered peptide would result in various possible positions for HD5 within the capsid depression (Figure 8). This heterogeneity in the HD5 position would explain why no cryoEM density was observed for HD5 in the HPV16+HD5 structure, even after filtering the HPV16+HD5 structure to 2 nm resolution (Figure 2).

## DISCUSSION

**Figure 9.**
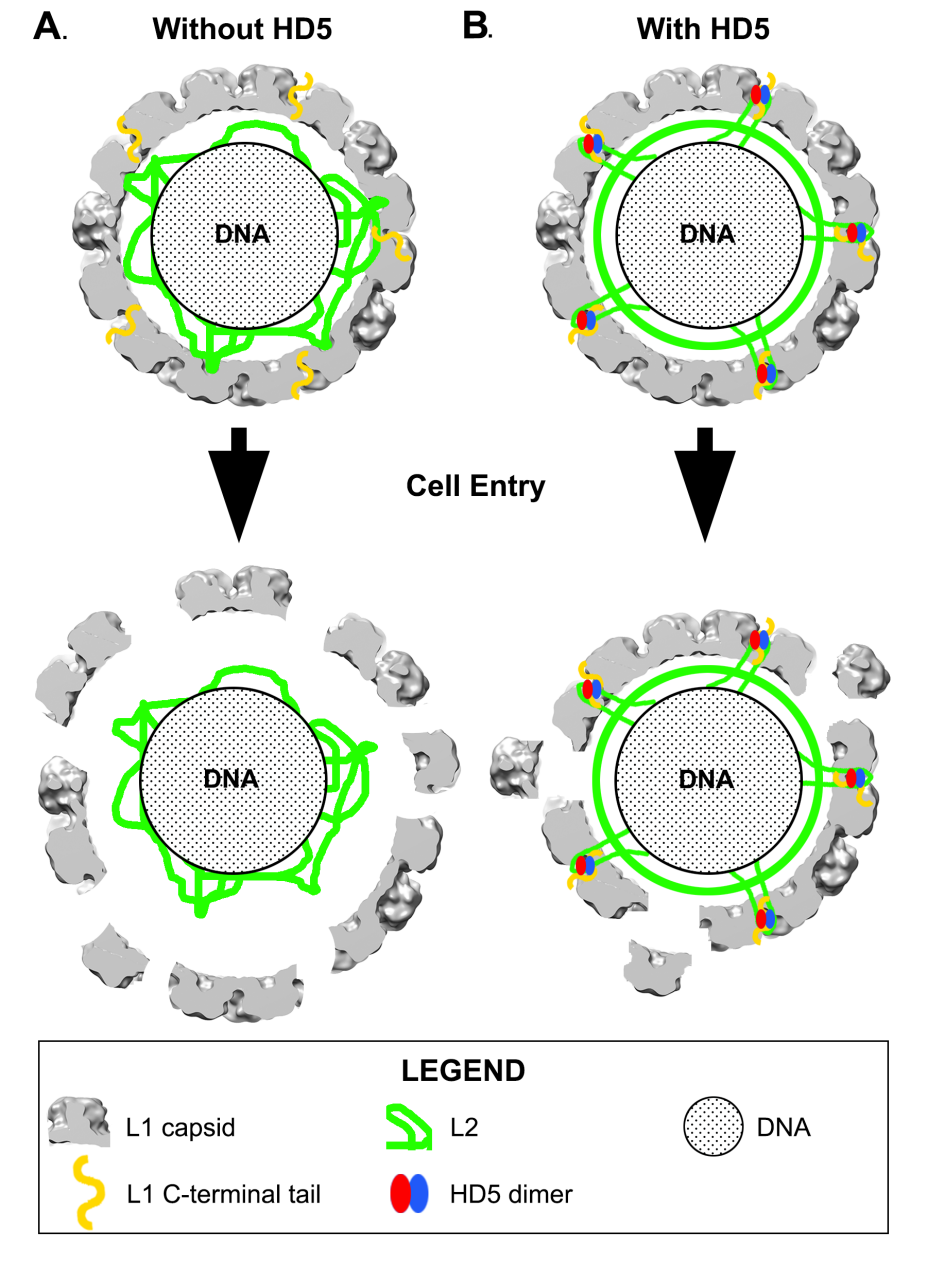
**Diagram illustrating proposed effect of HD5 on HPV16 structure and cell entry.** (A) In the absence of HD5, the ordered portion of the L1 capsid is represented by a central slab of the HPV16 cryo-EM density (gray), and some disordered L1 C-terminal tails (gold) are shown exposed on the exterior of the capsid. The disordered L2 protein (green) is represented as loosely associating with the L1 capsid and the encapsulated DNA genome (speckled region). After HPV16 enters the cell, the L1 protein dissociates from the L2/genome complex in the endosome. (B) In the presence of HD5, exposed L1 C-terminal tails are proposed to interact with HD5 dimers (red, blue) in depressions between L1 pentamers. In addition, negatively charged regions of L2 are proposed to be drawn through gaps in the L1 capsid and interact with HD5 dimers, effectively stabilizing the HPV16 capsid. After cell entry the virion is mostly held together by L1/L2/HD5 interactions.

Combining experimental information from cryoEM and mass spectrometry has enabled us to propose a model for how HD5 affects HPV16 structure and cell entry. The cryoEM results indicate that HD5 does *not* bind homogeneously to the well-ordered region of the L1 capsid. Nevertheless, the cryoEM results do show the effect of HD5 binding in stabilizing the capsid/core interaction with increased core density in the HPV16+HD5 cryoEM structure (Figure 2). The pseudoatomic structure of the L1 capsid [29] reveals openings in the capsid of ~1.5 nm in diameter. While these openings are too small to enable HD5 to enter the interior of the virion, they are large enough to allow disordered N- or C-terminal tails of L1 and portions of the disordered L2 protein to protrude through the capsid and interact with HD5. Thus, we propose that HD5 dimers are tethered within capsid depressions by disordered C-terminal tails of L1 and unidentified flexible or disordered regions of L2. The variable nature of HD5 tethering to the HPV16 capsid via disordered peptides would explain why no density is observed for HD5 in the subnanometer resolution cryoEM structure of HPV16+HD5 presented here (Figure 1). The proposed interconnection of L1 and L2 via HD5 may lead to increased ordering of L2 or the genome below the L1 capsid and would explain why more density is observed below the capsid in the cryoEM structure of HPV16+HD5 (Figure 2). In the absence of HD5, the L2/genome complex may be only loosely associated with the L1 capsid leading to separation of the L1 capsid and the L2/genome complex by the appropriate cellular triggers during cell entry [76] (Figure 9A). In the presence of HD5, regions of L2 may be so tightly associated with HD5 and the L1 capsid that separation of the L1 capsid and the L2/genome complex is impeded (Figure 9B). In our model, with HD5 present, the L1 capsid only partially uncoats during cell entry and the virion is mostly held together by L1/L2/HD5 interactions. This partial uncoating in the presence of HD5 would allow antibodies to access the genome (anti-BrdU) and internal epitopes of L1 exposed by cathepsin-mediated degradation (33L1-7 antibody) [16, 19]. Importantly, the hyper-stabilized interaction of L1 with the L2/genome complex leads to viral neutralization [16, 19].

Our model is consistent with both the new mass spectrometry data presented here as well as our prior biochemical and infectivity studies. For example, it offers a possible explanation for the ability of HD5 to prevent furin cleavage of L2 [18]. Localization of HD5 within capsid depressions may provide steric hindrance for the interaction of furin with the N-terminal tail of L2. Either HD5 prevents furin from approaching close enough to the capsid surface to effectively cleave the L2 N-terminal tail, or more likely, HD5 blocks N-terminal tails of L2 from protruding far enough to be cleaved by furin. As furin is significantly larger than an HD5 dimer, it seems likely that the L2 N-terminal tail would have to protrude above the capsid depressions in order to be cleaved by furin. The finding that furin-cleaved HPV16 is still sensitive to neutralization by HD5 [19] can also be explained by our HD5/HPV16 interaction model. Furin-cleaved HPV16 would be able to form the same interactions with HD5 as we have proposed for uncleaved HPV16. Therefore, the same hyper-stabilization of L1 with the L2/genome complex would be predicted to form with furin-cleaved HPV16, leading to viral neutralization.

Confirming which molecular components account for the density observed below the capsid in the cryoEM structure of HPV16+HD5 is challenging for multiple reasons. First, this density is not resolved at high resolution and corresponds to heterogeneously organized components. Second, cryoEM studies of L1-only virus-like particles (VLPs) would not be expected to inform our model, since it has been shown that L2 is required for DNA packaging in HPV16 [30, 31]. It is anticipated that structures of L1-only VLPs, either in the presence or absence of HD5, would not show any core density. In addition, the absence of L2 in L1-only VLPs may expose non-physiologic HD5 binding sites that are not present in mature virus or in PsV composed of L1 and L2. Thus, any additional density found in L1-only VLPs in the presence of HD5 may not be relevant to PsVs used in infection studies [16-20]. Previous studies have shown that HD5 protects both L1 and L2 from trypsin cleavage [19]. Although this prior study does not provide any direct evidence regarding where HD5 is binding to the capsid, it does suggest that both L1 and L2 are involved in the interaction with HD5. In previous cryoEM analyses of adenovirus neutralization by HD5, we proposed that HD5 interacts with a short region at the base of the fiber and a portion of the large, RGD-containing loop of penton base [23, 25]. In both the adenovirus and HPV16 cases, the structural models involve interactions of HD5 with disordered peptide regions, which makes HD5/virion interactions challenging to study by structural methods. In this report we show that mass spectrometry can add information that is complementary to cryoEM, and that the combination facilitates the generation of structural models. However, as indicated in Figure 8, several models and orientations of HD5 relative to L1 and L2 are consistent with the experimental data. Although more precision may be precluded by the dynamic nature of the capsid and its interactions with HD5, this model will guide future work to confirm which regions of HPV16 L1 and L2 are directly involved in binding HD5. These structural analyses will lead to a greater understanding of the mechanism of HD5 neutralization and the role of defensins in immunity to HPV and other viral infections.

## SUPPLEMENTARY MATERIALS

**Supplementary Table 1.** Mass spectrometry detected BS3 intra-protein crosslinks within L1

BS3 crosslinked residues	Band position on gel
L1(K475) - L1(K477)	Above L1 band, and L1/L2 dimer region
L1(K484) - L1(K486)	Above L1 band, and L1/L2 dimer region

**Supplementary Table 2.** Mass spectrometry detected BS3 intra-protein crosslinks within HD5-R13K/R32K

BS3 crosslinked residues	Band position on gel
HD5(K13) - HD5(K32)	Above L1 band, and above L2 band
